# Prevalence, risk factors and association with gallstone disease of non-alcoholic fatty liver disease among rural indigenous communities: A cross-sectional study in Sarawak, Malaysia

**DOI:** 10.51866/oa.634

**Published:** 2025-02-20

**Authors:** Ting Ting Yew, Whye Lian Cheah, Ai Jiun Koa, Han Bing Chow

**Affiliations:** 1 MD, Dr.Rad, FRCR, MMed, (Diagnostic Rad), Department of Radiology, Faculty of Medicine and Health Sciences, Universiti Malaysia Sarawak, Jalan Datuk Mohammad Musa, Kota Samarahan, Sarawak, Malaysia. Email: ttyew@unimas.my; 2 Phd (Community Nutrition), Department of Community Medicine & Public Health, Faculty of Medicine and Health Sciences, Universiti Malaysia Sarawak, Jalan Datuk Mohammad Musa, Kota Samarahan, Sarawak, Malaysia.; 3 MD, MMed Radiology, Department of Radiology, Faculty of Medicine and Health Sciences, Universiti Malaysia Sarawak, Jalan Datuk Mohammad Musa, Kota Samarahan, Sarawak, Malaysia.; 4 MBBCh BAO, Msc, MRCP, Department of Medicine, Faculty of Medicine and Health Sciences, Universiti Malaysia Sarawak, Jalan Datuk Mohammad Musa, Kota Samarahan, Sarawak, Malaysia.

**Keywords:** Non-alcoholic fatty liver disease, Fatty liver, Gallstone, Central obesity, Body mass index

## Abstract

**Introduction:**

This study aimed to evaluate the prevalence and risk factors of non-alcoholic fatty liver disease (NAFLD) among Dayak communities in Malaysia, shedding light on an underexplored population.

**Methods:**

A cross-sectional study was undertaken among Dayak villagers in Sarawak aged 18 years and above using an interview-based questionnaire, followed by an anthropometric measurement, a blood test and an abdominal ultrasound.

**Results:**

A total of 324 participants met the inclusion criteria. Among them, 42.9% were men, and the mean age was 49.85±14.9 years. The prevalence of NAFLD was substantially high at 58%, with 43.1% of the participants having mild fatty liver (grade 1). NAFLD was closely associated with waist circumference and body mass index (BMI) (P<0.001). Central obesity, as indicated by waist circumference and BMI, emerged as a potent risk factor, with higher values correlating with an increased likelihood of NAFLD. A higher prevalence of NAFLD was observed in the participants with an advancing age, an elevated triglyceride level (66.7%) and a lower high-density lipoprotein cholesterol level (81.6%). However, these associations did not remain significant in the multivariate analysis. Gallstones, which share common risk factors with NAFLD, were not significantly associated with NAFLD in this population (P=0.853).

**Conclusion:**

This study defines the prevalence and association of NAFLD with sociodemographic characteristics, health profiles and gallstone disease among indigenous villages in Dayak communities. A high BMI and central obesity are found to be independent risk factors of NAFLD.

## Introduction

Non-alcoholic fatty liver disease (NAFLD) has emerged as a worldwide health issue of alarming proportions, mirroring the rising trends of obesity and metabolic disturbances. This multifaceted liver disorder encompasses a range of pathological changes, from simple steatosis to non-alcoholic steatohepatitis, cirrhosis and hepatocellular carcinoma.^1^

Studies have shown that waist circumference, body mass index (BMI), age, hypertension, type 2 diabetes, triglyceride (TG) level and high- density lipoprotein cholesterol (HDL-C) level are the risk factors of NAFLD. Individuals with metabolic conditions, such as obesity, diabetes, hypertension and large waist circumference, tend to have a higher prevalence of NAFLD.^2^

In Malaysia, where rapid urbanisation and economic growth have precipitated substantial lifestyle changes, NAFLD has emerged as a significant issue within the nation’s healthcare landscape. With a prevalence ranging from 37.4% to 44.2%,^3,4^ it has become a prominent health concern, notably in individuals afflicted with metabolic syndrome (82.8%),^5^ dyslipidaemia (56.7%)^6^ and diabetes (49.6%).^7^

Despite the well-documented prevalence of NAFLD among certain Malaysian populations, there remains a paucity of resources and research efforts directed towards specific communities, particularly among the indigenous Dayak population of Borneo. Dayak people who primarily reside in the Southeast Asian regions of Borneo have distinctive dietary habits and lifestyles compared to other ethnicities in Malaysia. Dayak communities have their own unique cultural practices and dietary habits such as using local ingredients based on plants, including midin and fern, and applying special fermentation techniques in processing food, including tuak, an ethnic rice wine consumed as an alcoholic beverage during festivities or incorporated into meat dishes.^8^ These practices may serve as potential risk factors of both NAFLD and gallstone disease (GSD). While significant research has been conducted on NAFLD and GSD worldwide, there remains a considerable gap in knowledge regarding their potential coexistence and interplay, particularly within Dayak communities, which predominantly consume ethnic food. Limited studies have investigated this specific association in this ethnically diverse population in Borneo.

The associations between NAFLD and GSD in populations in the United States, China, Taiwan and Korea have been studied.^9-12^ NAFLD was associated with increased gallstone formation in studies on Chinese and Taiwanese populations,^10,11^ whereas inconclusive associations were found in studies on American and Korean populations owing to the association observed between NAFLD and cholecystectomy, rather than gallstones.^9,12^

The prevalence of NAFLD has surged in tandem, making it a pressing public health issue worldwide. This study sought to address this knowledge gap by uncovering the prevalence of NAFLD among Dayak communities in Malaysia and its intricate association with GSD. Understanding the unique dynamics of NAFLD within this population not only expands the knowledge but also serves as a stepping stone towards improving the quality of life and developing targeted interventions. This research serves as a critical step towards understanding the specific challenges posed by NAFLD in this population and illuminating the broader global context of NAFLD as a burgeoning health crisis.

## Methods

### Study design

A cross-sectional study was undertaken from September 2021 to June 2022. A list of Dayak villages in the Kuching, Serian and Samarahan divisions was obtained from the district officer, and villages were randomly selected from the list. An announcement was made via *Ketua Kampung* to invite all villagers aged 18 years and above to participate in the study. Villagers who were interested in participating in the study were required to fast for at least 8 hours the night before data collection on the next morning. An interview was conducted using a structured questionnaire that included sociodemographic questions, Alcohol Use Disorder Identification Test (AUDIT) items and medical history of chronic illnesses, followed by anthropometric measurement, blood pressure measurement, biochemical blood test and abdominal ultrasound. The AUDIT is a 10-item screening tool developed by the World Health Organization (WHO) to assess alcohol consumption, drinking behaviours and alcohol-related problems. Villagers with chronic liver disease, hepatitis B or C infection, a history of cholecystectomy, gallbladder tumour and heavy consumption of alcohol including *tuak* (AUDIT score of ≥8) were excluded. Hepatitis B or C status was based on self-disclosed status; no hepatitis blood screening or liver function test was carried out.

### Anthropometric measurement

Participants’ height was measured using the SECA 213 stadiometer and body weight using the SECA 813 weighing scale (Germany). Subsequently, BMI (kg/m^2^) was calculated. Based on the BMI classification in the WHO guidelines for the Asia-Pacific region,^13^ a BMI of <23 kg/m^2^ was classified as normal weight; 23.0-24.9 kg/m^2^, overweight; and ≥25 kg/m^2^, obesity.

Waist circumference was taken in the horizontal plane midway between the bottom edge of the last rib and the crest of the ilium using a nonelastic measuring tape. A waist circumference of ≥90 cm in men and ≥80 cm in women was considered to indicate central obesity.^14^

### Blood pressure measurement

Blood pressure was measured using a digital blood pressure monitor while participants were seated with their arms placed on the examination table and the cubital fossa supported at the heart level. Two readings were taken at a 1-minute interval, and the average of the two readings was used. Participants were categorised as hypertensive if their systolic blood pressure was ≥140 mmHg and/or diastolic blood pressure was ≥90 mmHg.^15^

### Biochemical measurement

Blood tests measured the fasting blood sugar (FBS), TG, total cholesterol (TC), HDL-C and low-density lipoprotein cholesterol (LDL-C) levels of participants. According to the 2020 Malaysian Diabetes Mellitus Guidelines and National Cholesterol Education Program- Adult Treatment Panel III, an FBS level of ≥5.6 mmol/L was considered to indicate hyperglycaemia,^16^ while a TG level of >1.7 mmol/L, a TC level of >5.2 mmol/L, an LDL-C level of >3.8 mmol/L or an HDL-C level of <1.0 mmol/L in men or <1.2 mmol/L in women was considered to indicate dyslipidaemia.^17^

### Diagnosis of NAFLD and gallstones

An abdominal ultrasound was conducted by two radiologists using the ultrasound machine Philips Portable High-Resolution Digital Colour Doppler Ultrasound System or General Electric Logic E, which is portable and has a 3.5-MHz convex probe. While participants were lying in the supine position, the liver was evaluated via the sagittal view, comparing the right lobe of the liver and right kidney, transverse view on the left lobe of the liver and spleen and transverse view on the liver and pancreas. Fatty liver was diagnosed if at least two out of the following three criteria were met: 1) increased liver echogenicity compared to the renal cortex and spleen, 2) attenuation of the ultrasound wave and loss of definition of the diaphragm and 3) impaired delineation of the intrahepatic vessels. The severity of echogenicity was assessed using the semiquantitative visual grading system as follows: mild or grade 1 if there was a slight, diffuse increase in fine echoes in the liver parenchyma, with normal visualisation of the diaphragm and intrahepatic vessel borders; moderate or grade 2 if there was a moderate, diffuse increase in fine echoes, with slightly impaired visibility of the intrahepatic vessels and diaphragm; and severe or grade 3 if there was a marked increase in fine echoes, with poor or no visibility of the intrahepatic vessel borders, diaphragm and posterior right lobe of the liver.^18^

Gallstones were diagnosed after participants fasted for at least 6 hours, with the following criteria: 1) a highly reflective echo from the anterior surface of the gallstone, 2) mobility of the gallstone with positioning (typically in the decubitus position) and 3) marked posterior acoustic shadowing.^19^

### Statistical analysis

The collected data were analysed using the Statistical Package for the Social Sciences version 25 to generate descriptive data and determine the risk factors of NAFLD. Categorical and continuous variables were presented as percentages and means with standard deviations, respectively. The chi-square test was conducted for comparisons between proportions and a t-test for comparisons of means between two groups. A P-value less than 0.05 was considered statistically significant. Multivariate analysis was conducted using multiple logistic regression to determine the predictive risk factors of NAFLD. Odds ratios (ORs) were used to quantify the strength of the association of particular factors with NAFLD, along with 95% confidence intervals.

## Results

A total of 324 participants (139 men and 185 women), with a mean age of 49.85±14.9 years, were included in the study. Among them, 91.0% identified as Bidayuh, while 9.0% identified as Iban. The majority were married (77.8%) and possessed a level of education (93.3%). Additional details concerning the sociodemographic profiles of the participants are presented in Table 1.

**Table 1:** Sociodemographic characteristics of the participants (N=324).

	n (%)	Mean (SD)
Age (year)		49.85 (14.9)
**Sex**		
Male	139 (42.9)	
Female	185 (57.1)	
**Race**		
Bidayuh	295 (91.0)	
Iban	29 (9.0)	
**Marital status**		
Single	72 (22.3)	
Married	252 (77.8)	
**Educational level**		
No education	15 (4.6)	
Primary school	43 (13.3)	
Secondary school	154 (47.5)	
Diploma	53 (16.4)	
University	52 (16.0)	
Missing data	7 (2.1)	

Table 2 depicts the health profile of the participants. Elevated blood sugar levels were observed in a substantial number of participants (71.6%), with an average level of 6.37 mmol/L. A significant proportion (83.4%) were classified as overweight and obese, with an average BMI of 27.37 kg/m^2^. Furthermore, 53.7% of the participants exhibited a large waist circumference. Conversely, more than half showed normal TG (54.6%), HDL-C (88.6%) and LDL-C levels (72.8%) and blood pressure (67.3%). Regarding the liver ultrasound results, 58% of the participants were diagnosed with NAFLD. Within this NAFLD group, 24.9% had mild fatty liver (grade 1); 19.3% had moderate fatty liver (grade 2); and 13.8% had severe fatty liver (grade 3). Conversely, only 6% of the entire study sample was found to have gallstones.

**Table 2:** Biochemical and anthropometric measurements of the participants (N=324).

Variable	n (%)	Mean (SD)
FBS level (mmol/L)		6.37 (1.89)
Normal (<5.6)	92 (28.4)	
High (≥5.6)	232 (71.6)	
TG level (mmol/L)		1.88 (0.90)
Normal (≤1.7)	177 (54.6)	
High (>1.7)	147 (45.4)	
HDL-C level (mmol/L)		1.47 (0.36)
Normal	286 (88.3)	
Low (<1.0 in men and <1.2 in women)	38 (11.7)	
LDL-C level (mmol/L)		3.39 (0.94)
Normal	236 (72.8)	
High (>3.8)	88 (27.2)	
Total cholesterol level (mmol/L)		5.54 (1.03)
Normal	144 (44.4)	
High (>5.2)	180 (55.6)	
BMI (kg/m^2^)		5.54 (1.03)
Underweight (<18.5)	10 (3.1)	
Normal (18.5-22.9)	44 (13.6)	
Overweight (≥23)	123 (38.0)	
Obese (≥27.5)	147 (45.4)	
Waist circumference (cm)		85.59 (11.79)
Normal	150 (46.3)	
Large (≥80 cm in women and ≥90 cm in men)	174 (53.7)	
**Blood pressure (mmHg)**		
Normal	218 (67.3)	
High (SBP of ≥140 and/or DBP of ≥90)	106 (32.7)	
**NAFLD**		
No	136 (42.0)	
Mild (grade 1)	81 (25.0)	
Moderate (grade 2)	63 (19.4)	
Severe (grade 3)	44 (13.6)	
**Gallstone**		
No	304 (93.8)	
Yes	20 (6.2)	

FBS: fasting blood sugar, TG: triglyceride, HDL-C: high-density lipoprotein cholesterol, LDL-C: low-density lipoprotein cholesterol, BMI: body mass index

The association between NAFLD and its influencing factors is outlined in Table 3. Age demonstrated a significant correlation with NAFLD (P<0.001). In terms of the anthropometric measurements, a noteworthy link between BMI and NAFLD was established. The prevalence of NAFLD substantially increased with increasing BMI (P<0.001), ranging from 22.7% among those with a normal BMI to 52% and 77.6% among the overweight and obese participants, respectively. Similarly, a large waist circumference exhibited a significant correlation with NAFLD (P<0.001), with the participants with a large waist circumference having a higher prevalence of NAFLD than those with a normal waist circumference. Concerning the biochemical measurements, a significantly elevated prevalence of NAFLD was identified among the participants with a high TG level (66.7%) and a low HDL-C level (81.6%) in comparison to those with normal TG and HDL-C levels (50.8% and 54.9%; P=0.004 and 0.002, respectively). Nonetheless, no significant associations were observed between sex, race, FBS level, LDL-C level, TC level, blood pressure, gallstones and NAFLD.

**Table 3:** Comparison of the biochemical and anthropometric measurements of the participants with and without NAFLD (N=324).

Variable	Without NAFLD, n (%)	With NAFLD, n (%)	P-value
**Age (mean)**	49.1	50.3	<0.001
**Sex**			0.760
Male	57 (41.0)	82 (59.0)	
Female	79 (42.7)	106 (57.3)	
Race			0.744
Bidayuh	123 (41.7)	172 (58.3)	
Iban	13 (44.8)	16 (55.2)	
FBS level (mmol/L)			0.065
Normal (<5.6)	46 (50.0)	46 (50.0)	
High (≥5.6)	90 (33.8)	142 (75.5)	
TG level (mmol/L)			0.004
Normal (≤1.7)	87 (49.2)	90 (50.8)	
High (>1.7)	49 (33.3)	98 (66.7)	
HDL-C level (mmol/L)			0.002
Normal	129 (45.1)	157 (54.9)	
Low (<1.0 in men and <1.2 in women)	7 (18.4)	31 (81.6)	
LDL-C level (mmol/L)			0.200
Normal	94 (39.8)	142 (60.2)	
High (>3.8)	42 (47.7)	46 (52.3)	
Total cholesterol level (mmol/L)			0.217
Normal	55 (38.2)	89 (61.8)	
High (>5.2)	81 (45.0)	99 (55.0)	
BMI (kg/m^2^)			<0.001
Underweight (<18.5)	10 (100.0)	0 (0.0)	
Normal (18.5-22.9)	34 (77.3)	10 (22.7)	
Overweight (≥23)	59 (48.0)	64 (52.0)	
Obese (≥27.5)	33 (22.4)	114 (77.6)	
Waist circumference (cm)			<0.001
Normal	91 (60.7)	59 (39.3)	
Large (≥80 cm in women and ≥90 cm in men)	45 (25.9)	129 (74.1)	
Blood pressure (mmHg)			0.072
Normal	99 (45.4)	119 (54.6)	
High (SBP of ≥140 and/or DBP of ≥90)	37 (34.9)	69 (65.1)	
Gallstone			0.853
No	128 (42.1)	176 (57.9)	
Yes	8 (42.0)	12 (58.0)	

FBS: fasting blood sugar, TG: triglyceride, HDL-C: high-density lipoprotein cholesterol, LDL-C: low-density lipoprotein cholesterol, BMI: body mass index, SBP: systolic blood pressure, DBP: diastolic blood pressure

The results of the multiple logistic regression analysis of the risk factors of NAFLD are displayed in Table 4. Both BMI and waist circumference significantly contributed to the development of NAFLD. A higher BMI and a larger waist circumference were associated with increased odds of NAFLD. The Wald values for the independent variables indicated that only BMI and waist circumference served as significant predictors for NAFLD. Notably, age, sex, race, FBS level, TG level, HDL-C level, LDL-C level, TC level, blood pressure and gallstones did not exhibit significant associations with NAFLD.

**Table 4:** Multivariate analysis of the risk factors of NAFLD.

	B	SE	Wald	df	Sig.	Exp(B)	95% CI for Exp(B)	
							Lower	Upper
Age	0.002	0.011	0.026	1	0.873	1.002	0.980	1.024
Sex	0.475	0.315	2.277	1	0.131	1.608	0.868	2.979
Race	0.134	0.453	0.088	1	0.767	1.144	0.471	2.776
FBS level	0.162	0.118	1.901	1	0.168	1.176	0.934	1.481
TG level	0.713	0.424	2.830	1	0.093	2.039	0.889	4.679
HDL-C level	0.272	0.862	0.100	1	0.752	1.313	0.243	7.104
LDL-C level	1.096	0.897	1.491	1	0.222	2.991	0.515	17.362
Total cholesterol level	-1.068	0.870	1.506	1	0.220	0.344	0.062	1.892
BMI	0.132	0.051	6.681	1	0.010	1.142	1.033	1.262
Waist circumference	0.049	0.022	4.994	1	0.025	1.050	1.006	1.096
SBP	-0.007	0.013	0.289	1	0.591	0.993	0.969	1.018
DBP	0.029	0.019	2.401	1	0.121	1.030	0.992	1.069
Gallstone	-0.330	0.550	0.360	1	0.549	0.719	0.245	2.113
Constant	-10.221	2.099	23.721	1	0.000	0.000		

SE: standard error, df: degree of freedom, Sig: significant P, Exp(B): odds ratio, CI: confidence interval, FBS: fasting blood sugar, TG: triglyceride, HDL-C: high-density lipoprotein cholesterol, LDL-C: low-density lipoprotein cholesterol, BMI: body mass index, SBP: systolic blood pressure, DBP: diastolic blood pressure

## Discussion

The current study investigated the relationship between NAFLD and various demographic, anthropometric and biochemical factors within a cohort of 324 participants. This study uncovered a high prevalence of NAFLD at 58% and a significant association with waist circumference and BMI (P<0.001). Notably, gallstones, which share common risk factors with NAFLD, did not show a significant association with NAFLD in this particular population.

The prevalence of NAFLD in our study sample was noteworthy, with 58% of the participants diagnosed based on liver ultrasound findings. This prevalence is alarmingly higher than that in other states in Malaysia (e.g. 37.4% among the urban population in Klang Valley^3^ and 19.6% among the rural indigenous population in Peninsular Malaysia^20^). It also surpasses the prevalence reported in other world regions such as 26.9% in Europe^21^ and 29.62% in Asia.^22^ The majority of the participants in our study had mild NAFLD (grade 1), which is similar to other studies among other ethnic groups in Malaysia.^23^ The concerning uptick in the prevalence of NAFLD can mainly be ascribed to changes in diet and lifestyle prompted by rapid economic growth and urbanisation.^24^ This finding aligns with the growing recognition of NAFLD as a significant health concern, particularly caused by its association with obesity and metabolic disturbances.

The observed distribution of NAFLD severity, ranging from mild to severe fatty liver changes, underscores the need for comprehensive assessments of disease progression and management strategies tailored to individual profiles. Lifestyle interventions such as dietary therapy and exercise have demonstrated benefits in reducing the risk of NAFLD. High-fibre, low-fat, low-carbohydrate, hypocaloric and high- protein diets are the dietary measures suggested to control liver disease activities and risk factors.^25^

The anthropometric measurements, particularly BMI and waist circumference, exhibited significant associations with NAFLD. A higher BMI and a larger waist circumference (central obesity) were strongly linked to an elevated risk of NAFLD, as indicated by their significant ORs in the logistic regression analysis. The prevalence of NAFLD increases almost linearly with BMI, as shown in Korean populations.^26^ These findings are consistent with other reports, in which the prevalence of NAFLD in obese individuals was higher than that in the general population.^27^ In obesity-driven NAFLD, adipose tissue expansion, dysfunction, insulin resistance and inflammation lead to fat accumulation in the liver. Lipotoxicity, glucotoxicity and fibrogenesis contribute to NAFLD progression and potentially culminate in cirrhosis due to impaired tissue regeneration.^28^ This emphasises the central role of obesity in the pathogenesis of NAFLD and highlights the importance of weight management strategies in NAFLD prevention and control. Weight loss of ≥10% from lifestyle modifications significantly reduced the NAFLD activity score in 90% and improved liver fibrosis in 45%–81% of participants in the study by Vilar-Gomez et al.^29^ However, other demographic factors such as age, sex and race did not exhibit significant associations with NAFLD in our study, indicating that NAFLD’s impact might transcend these factors.

In terms of the biochemical measurements, elevated TG levels and low HDL-C levels were associated with a higher prevalence of NAFLD in the univariate analysis. These findings are consistent with many previous reports^30^ indicating that hypertriglyceridaemia and low HDL-C levels were the risk factors for developing NAFLD. This relationship emphasises the intricate interplay between lipid metabolism and liver health, where dyslipidaemia may contribute to the progression of hepatic steatosis. It is still an ongoing debate whether the fat accumulation within hepatocytes causes lipid metabolism abnormalities or whether lipid metabolism abnormalities trigger the development of NAFLD. Nevertheless, the multivariate logistic regression analysis revealed that the TG and HDL-C levels did not emerge as significant risk factors. This may be because Dayak communities consume foods mainly consisting of natural ingredients,^8^ thus reducing the risks of developing NAFLD. The present findings are consistent with the report by Malik et al., wherein the TG, HDL-C, LDL-C and TC levels were not found to be predictors for NAFLD,^23^ although Goh et al. found the TG and low LDL-C levels as predictors for NAFLD.^30^ Conversely, our study showed no significant associations between the FBS level, LDL-C level, blood pressure and NAFLD. Similarly, other studies demonstrated no significant association between diabetes, hypertension and NAFLD.^31^

In this study, gallstones were not significantly associated with NAFLD, although both NAFLD and GSD share the same risk factors, including insulin resistance, obesity, metabolic syndrome and type 2 diabetes mellitus. This suggests that these two conditions might have distinct underlying mechanisms and pathogeneses.

The study has several limitations. First, the abdominal ultrasounds were conducted using two sonography machines, potentially resulting in varying grades of NAFLD due to differences in machine echo attenuation quality. Second, this study did not evaluate the potential effect of lifestyle factors such as dietary and exercise habits, which may have an impact on the development of NAFLD. Third, the new nomenclature of metabolic-associated fatty liver disease has been recently suggested; however, this study could not apply this concept, as there was an absence of data on plasma high-sensitivity C-reactive protein and fasting insulin levels.

## Conclusion

Our study provides valuable insights into the prevalence and risk factors of NAFLD in the Borneo Dayak population. The high prevalence of NAFLD highlights the urgency of public health initiatives aimed at addressing obesity and its associated metabolic sequelae. The significant associations observed for age, BMI and waist circumference emphasise the need for comprehensive assessment and intervention strategies tailored to individual profiles. Lifestyle modifications including dietary change, exercise and weight loss are suggested to reduce the development of NAFLD. However, further long-term studies are warranted to elucidate causal relationships and guide the development of targeted interventions for NAFLD prevention and management.

## Data sharing statement

The datasets generated during the current study are available from the corresponding author upon reasonable request.

**How does this paper make a difference in general practice?**

- Given the high prevalence of non-alcoholic fatty liver disease (NAFLD) in Malaysia, this study demonstrates the critical need to raise awareness among healthcare providers about screening and early management of NAFLD.
- This study identifies the risk factors of NAFLD, including a high body mass index, a large waist circumference and dyslipidaemia.
- Understanding the prevalence and risk factors of NAFLD allows general practitioners to tailor management strategies and targeted interventions such as lifestyle modifications, dietary counselling and weight management.

## References

[1] Ozturk ZA, Kadayifci A (2014). Insulin sensitizers for the treatment of non-alcoholic fatty liver disease.. World J Hepatol..

[2] National Guideline Centre (UK). (2016). Risk Factors for NAFLD..

[3] Khammas ASA, Hassan HA, Salih SQM (2018). Prevalence and risk factors of sonographically detected non alcoholic fatty liver disease in a screening centre in Klang Valley, Malaysia: an observational cross-sectional study.. Porto Biomed J..

[4] Cheah WL, Lee PY, Chang CT, Mohamed HJ, Wong SL (2013). Prevalence of ultrasound diagnosed nonalcoholic fatty liver disease among rural indigenous community of Sarawak and its association with biochemical and anthropometric measures.. Southeast Asian J Trop Med Public Health..

[5] Suppiah S, Lee RC, Sazali NS, Hassan H (2016). Non-alcoholic fatty liver disease in metabolic syndrome patients in Serdang Hospital: quantification by contrast-enhanced computed tomography.. Faculty of Medicine and Health Sciences..

[6] Magosso E, Ansari MA, Gopalan Y (2010). Prevalence of non-alcoholic fatty liver in a hypercholesterolemic population of northwestern peninsular Malaysia.. Southeast Asian J Trop Med Public Health..

[7] Chan WK, Tan AT, Vethakkan SR, Tah PC, Vijayananthan A, Goh KL (2013). Non-alcoholic fatty liver disease in diabetics--prevalence and predictive factors in a multiracial hospital clinic population in Malaysia.. J Gastroenterol Hepatol..

[8] Ting H, Tan SR, John AN (2017). Consumption intention toward ethnic food: determinants of Dayak food choice by Malaysians.. J Ethn Foods..

[9] Ruhl CE, Everhart JE (2013). Relationship of nonalcoholic fatty liver disease with cholecystectomy in the US population.. Am J Gastroenterol..

[10] Liu J, Lin H, Zhang C (2014). Non-alcoholic fatty liver disease associated with gallstones in females rather than males: a longitudinal cohort study in Chinese urban population.. BMC Gastroenterol.

[11] Lee YC, Wu JS, Yang YC, Chang CS, Lu FH, Chang CJ (2014). Moderate to severe, but not mild, nonalcoholic fatty liver disease associated with increased risk of gallstone disease.. Scand J Gastroenterol..

[12] Kwak MS, Kim D, Chung GE, Kim W, Kim YJ, Yoon JH (2015). Cholecystectomy is independently associated with nonalcoholic fatty liver disease in an Asian population.. World J Gastroenterol..

[13] International Association for the Study of Obesity, International Obesity Task Force (IOTF). (2000). The Asia-Pacific Perspective: Redefining Obesity and Its Treatment..

[14] Task Force on Epidemiology and Prevention. (2006). The IDF Consensus Worldwide Definition of the Metabolic Syndrome..

[15] Clinical Practice Guidelines Management of Hypertension. (2018).

[16] Clinical Practice Guidelines Management of Type 2 Diabetes Mellitus. (2020).

[17] Clinical Practice Guidelines Management of Dyslipidemia. (2017).

[18] Hamer OW, Aguirre DA, Casola G, Lavine JE, Woenckhaus M, Sirlin CB (2006). Fatty liver: imaging patterns and pitfalls.. Radiographics..

[19] Murphy MC, Gibney B, Gillespie C, Hynes J, Bolster F (2020). Gallstones top to toe: what the radiologist needs to know.. Insights Imaging..

[20] Kirovski G, Schacherer D, Wobser H (2010). Prevalence of ultrasound-diagnosed nonalcoholic fatty liver disease in a hospital cohort and its association with anthropometric, biochemical and sonographic characteristics.. Int J Clin Exp Med..

[21] Cholongitas E, Pavlopoulou I, Papatheodoridi M (2021). Epidemiology of nonalcoholic fatty liver disease in Europe: a systematic review and metaanalysis.. Ann Gastroenterol..

[22] Li J, Zou B, Yeo YH (2019). Prevalence, incidence, and outcome of non-alcoholic fatty liver disease in Asia, 1999-2019: a systematic review and meta-analysis.. Lancet Gastroenterol Hepatol..

[23] Malik A, Cheah PL, Hilmi IN, Chan SP, Goh KL (2007). Non-alcoholic fatty liver disease in Malaysia: a demographic, anthropometric, metabolic and histological study.. J Dig Dis..

[24] Fan JG, Kim SU, Wong VW (2017). New trends on obesity and NAFLD in Asia.. J Hepatol..

[25] Ahn J, Lee HY, Moon JH (2019). Critical appraisal for low-carbohydrate diet in nonalcoholic fatty liver disease: review and metaanalyses.. Clin Nutr..

[26] Chang Y, Jung HS, Cho J (2016). Metabolically healthy obesity and the development of nonalcoholic fatty liver disease.. Am J Gastroenterol..

[27] Li L, Liu DW, Yan HY, Wang ZY, Zhao SH, Wang B (2016). Obesity is an independent risk factor for non-alcoholic fatty liver disease: evidence from a meta-analysis of 21 cohort studies.. Obes Rev..

[28] Polyzos SA, Kountouras J, Mantzoros CS (2019). Obesity and nonalcoholic fatty liver disease: from pathophysiology to therapeutics.. Metabolism..

[29] Gomez E, Perez Y, Calzadilla-Bertot L (2015). Weight loss through lifestyle modification significantly reduces features of nonalcoholic steatohepatitis.. Gastroenterology..

[30] Goh SC, Ho EL, Goh KL (2013). Prevalence and risk factors of non-alcoholic fatty liver disease in a multiracial suburban Asian population in Malaysia.. Hepatol Int..

[31] Almobarak AO, Barakat S, Khalifa MH, Elhoweris MH, Elhassan TM, Ahmed MH (2014). Non alcoholic fatty liver disease (NAFLD) in a Sudanese population: what is the prevalence and risk factors?.. Arab J Gastroenterol..

